# Converting fibroblastic fates leads to wound healing without scar

**DOI:** 10.1038/s41392-021-00738-6

**Published:** 2021-09-01

**Authors:** Dongsheng Jiang, Yuval Rinkevich

**Affiliations:** 1 Institute of Lung Biology and Disease, Helmholtz Zentrum München, Munich, Germany; 2 Institute of Regenerative Biology and Medicine, Helmholtz Zentrum München, Munich, Germany

**Keywords:** Cell biology, Developmental biology, Physiology

In a recent paper published in *Science*, Mascharak et al.^1^ demonstrated that inhibiting Engrailed-1 activation in wound fibroblasts promotes scarless wound repair that phenocopies wound repair by regeneration seen in lower taxon groups and rarely in mammals.

Many living species of chordates, including vertebrates, respond to injury by regenerating a perfect tissue replica. Mammals and humans on the other hand, with some rare exceptions, replaced true regeneration with scar tissue build-up in wounds. Scars form a quick seal that closes open wounds, and severely restrict tissue biomechanics, function and physiological performance. The mechanistic choice of wounded mammalian tissues to respond by either scarring or regenerating holds tremendous therapeutic potential for preserving and restoring the functions of injured tissues, but remains a biological puzzle.

The skin serves a prototypical model to explore the underlying mechanisms of scarring and regeneration, because both scarring and regeneration of skin are seen across developmental time, diverse anatomic locations, and injury depths. The current existing cellular resolution of fibroblastic stromal cells in the skin indicates that functionally diverse embryonic lineages of fibroblasts co-inhabit the skin, and to a large degree dictate the skin’s diverging scarring and regeneration responses. In the backskin, two functionally distinct fibroblastic lineages were identified, based on the expression history of *Engrailed-1* (*En1*). *En1* is a homeobox transcription factor, which was thought to be expressed only at the early stages of embryonic development. En1 lineage-positive fibroblasts (EPFs) refers to the progeny of fibroblastic ancestors that had expressed *En1* during early embryonic development. Adult EPFs have been demonstrated to be the fibroblastic lineage responsible for connective tissue build up in multiple fibrotic skin models, including excisional wound models and in melanoma tumour stroma formation. Whereas En1 lineage-negative fibroblasts (ENFs) are a separate lineage of skin fibroblasts that does not share a history of *En1* expression, and in wounds refrain from contributing to fibrotic responses.^2^ EPFs and ENFs thus show lineage-specific functions as two specialized adult cell types.

Mascharak et al.^1^ have challenged this notion, and called lineage-restriction into question, by proposing that fully committed ENF fibroblasts can respond to tissue challenges, such as injury, by acquiring new fates and converting to EPFs. This phenotypic adaptability is termed plasticity. The authors show that in adult wounds *En1* can be reactivated postnatally in ENFs via mechanotransduction signalling. The converted new EPFs are called postnatal EPFs (pEPFs), differentiating them from embryonic EPFs (eEPFs). The authors argue that turning off En1 blocks the ENF-to-pEPF conversion and is sufficient for skin regeneration with regrowth of skin apendages.^1^

Using tamoxifen-inducible Cre mouse lines (*En1*^*Cre-ERT*^*;Ai6* and *En1*^*Cre*^*;R26*^*mTmG*^) coupled with splinted full-thickness excisional wound models, the authors precisely traced En1-lineage during wound healing processes, and revealed that ~40% of wound fibroblasts were pEPFs. Next, the authors utilized a tension loaded incisional wound model and tissue culture assays to show that mechanical tension significantly increased ENF-to-pEPFs conversion, and was correlated with elevated number of cells expressing Yes-associated protein (YAP) in nuclei, the key effector of the mechanotransduction pathway. The authors further showed that pharmacological inhibition of YAP by verteporfin, or diphtheria toxin-mediated targeted ablation of pEPFs in *En1*^*Cre-ERT*^*;Ai6;R26*^*iDTR*^ wounds culminates in skin regeneration 30 days after wounding. To rule out the off-target effect of verteporfin, further investigation was carried in a pEPF-specific YAP knockout models (*En1*^*Cre-ERT*^*;R26*^*mTmG*^*;YAP*^*fl/fl*^). Almost complete abolishment of ENF-to-pEPF conversion and the same regenerative phenotype was observed.

**Fig. 1:**
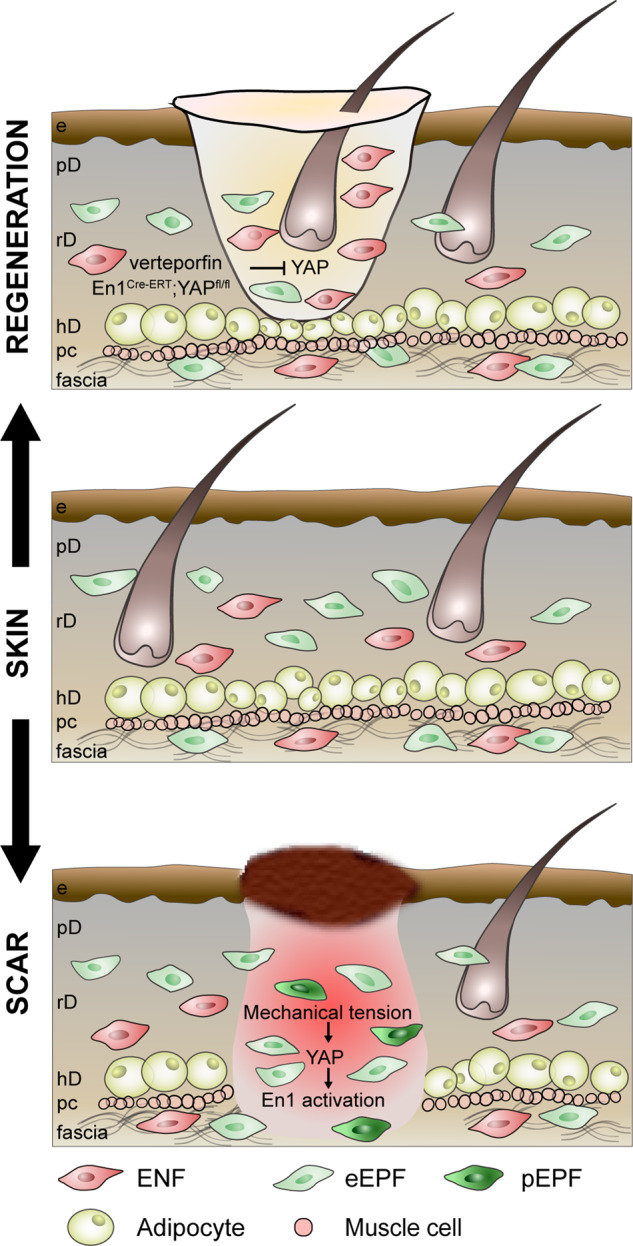
Mechanical tension in wounds activates YAP signalling in ENFs, leading to postnatal activation of *En1*. The conversion of ENFs to pEPFs contributes to skin scarring. Inhibition of mechanotransduction regulator YAP by chemical inhibitor verteprofin or pEPF-specific YAP knockout prevents the conversion and leads to skin regeneration. e epidermis, pD papillary dermis, rD reticular dermis, hD hypodermis, pc panniculus carnosus, eEPF embryonic EPF, pEPF postnatal EPF

Previously, EPFs and ENFs were thought to remain completely segregated after shutdown of *En1* expression during early embryogenesis, and to retain lineage-specific functions. This study indicates that ENFs, via sensing the mechanical stress in the wound environment, acquire *En1* transcript expression and become scar-forming EPFs, which in turn magnify the scarring outcome. The potential to inhibit the interconversion between a non-scarring to a scarring stromal lineage (ENFs-to-EPFs), holds tremendous potential for scarless wound repair. It may further provide a novel therapeutic tactic to ameliorate fibrotic responses and induce regeneration, by modulating biomechanical properties of the wound microenvironment or manipulating mechanotransduction in wound fibroblasts (Fig. 1).

Despite providing evidence that biomechanical forces influence the pattern of regulation of *En1* transcription, a formal proof of the role of EN1 in scar formation remains to be determined. This is especially relevant with fibroblasts, because 2D cell culture or long-lasting aberrant tension alters transcriptomic, proteomic and surface marker profiles across fibroblasts, regardless of their intrinsic lineage property. Moreover, ENFs in adult skin comprise a minority (<20%) of fibroblasts across all dermal layers.^2,3^ Embryonic EPFs should therefore outnumber pEPFs by 4 to 1, even if all adult ENFs convert to pEPFs. Furthermore, EPFs have been shown to be enriched in wounds by collective cell migration that is mediated by intercellular adhesion molecule N-cadherin upregulated specifically on EPFs.^4^ Therefore, it remains surprising that prevention of ENF-to-pEPF conversion is sufficient to induce regeneration, despite eEPFs being present in wounds and sharing the same profibrotic functions as pEPFs.^1^

Mascharak et al. show that ∼40% wound fibroblasts are pEPFs, a percentage that is not reachable via ENF conversion alone. This raises further questions as to whether ENFs include a subset of unipotent fibroblast progenitors that can differentiate into ENFs and EPFs? Is the conversion of wound ENFs to EPFs a differentiation process accompanied with proliferation or with N-Cadherin reactivation? Can this differentiation process be reversed after taking place? Does *En1* need to be reactivated in eEPFs for their scarring functions and what is the role of EN1 in this phenotypic conversion?

Third, the treatment of YAP inhibitor verteporfin in wounds of non-inducible Cre lines (*En1*^*Cre*^*;R26*^*mTmG*^) resulted in substantial reduction of EPFs, including eEPFs that were ∼60% in wounds. Does inhibition of YAP deplete eEPFs or does it induce a conversion back to ENFs? A transition that would not be noticeable in a Cre-mTmG system, because once GFP starts expression upon Cre recombinase, tdTomato transgene is deleted, shutting down tdTomato protein expression. Importantly, the authors did not address the question of what is the exact functional consequences of En1 and of YAP in ENFs. High nuclear YAP was found in ENFs, but knockout of YAP specifically in pEPFs with *En1*^*Cre-ERT*^*;YAP*^*fl/fl*^ transgenic mice leads to regeneration. Is YAP a critical upstream regulator of ENF-to-pEPF conversion, or of EPFs? How does mechanical tension differentially affect ENFs and EPFs to induce formations of two alternative connective tissue structures as in scarring and regeneration also remains an important question clinically requiring further investigation.

Fourth, how does *En1* transcription activation turns on the matrix remodelling functions in wound fibroblasts remains unknown. The exact downstream target genes of EN1 and the exact function of EN1 in controlling fibrogenicity remains undetermined. The answer to this question may provide additional insights on whether a silencing strategy against EN1 in wound fibroblasts can be used as an alternative approach for skin regeneration.

A recent study demonstrates that the subcutaneous fascia is critical for the early repair of deep wounds and the subsequent scarring event.^5^ The fascia fibroblasts have a similar ENF-EPF composition as in reticular dermis, but reside in a distinct viscoelastic jelly-like ECM environment with pre-made matrix fibres. Both excisional and incisional wound models used in the study of Mascharak et al. are full-thickness wounds, which certainly involve subcutaneous fascia. In fact, many YAP^+^ cells are present in deep wound layers, indicating their fascia origin. Considering that fascia is the tissue specialized in sensing mechanical stress/load, it is plausible that in addition to the reticular dermal fibroblasts, fascia fibroblasts are involved in scarring with the newly discovered mechanical sensing and ENF-EPF conversion mechanisms.

Last but not least, for imperative translational purposes, an important question remains about whether equivalent cells of ENFs and EPFs exist in human skin. The precise expression patterns of En1 on various human fibroblast subtypes are not well defined and need to be elucidated.

With these questions and perspectives in mind, further investigation building on the basis of the work from Mascharak et al. and others would lead to achieve the ultimate goal of a scarless wound healing.
